# Limited Internal Comparability of General Intelligence Composites: Impact on External Validity, Possible Predictors, and Practical Remedies

**DOI:** 10.1177/10731911211005171

**Published:** 2021-04-02

**Authors:** Silvia Grieder, Anette Bünger, Salome D. Odermatt, Florine Schweizer, Alexander Grob

**Affiliations:** 1 University of Basel, Basel, Switzerland

**Keywords:** general intelligence, IQ, screening, individual level, reliability, validity

## Abstract

Research on comparability of general intelligence composites (GICs) is scarce and has focused exclusively on comparing GICs from different test batteries, revealing limited individual-level comparability. We add to these findings, investigating the group- and individual-level comparability of different GICs within test batteries (i.e., internal score comparability), thereby minimizing transient error and ruling out between-battery variance completely. We (a) determined the magnitude of intraindividual IQ differences, (b) investigated their impact on external validity, (c) explored possible predictors for these differences, and (d) examined ways to deal with incomparability. Results are based on the standardization samples of three intelligence test batteries, spanning from early childhood to late adulthood. Despite high group-level comparability, individual-level comparability was often unsatisfactory, especially toward the tails of the IQ distribution. This limited comparability has consequences for external validity, as GICs were differentially related to and often less predictive for school grades for individuals with high IQ differences. Of several predictors, only IQ level and age were systematically related to comparability. Consequently, findings challenge the use of overall internal consistencies for confidence intervals and suggest using confidence intervals based on test–retest reliabilities or age- and IQ-specific internal consistencies for clinical interpretation. Implications for test construction and application are discussed.

General intelligence is defined as the broad mental capacity to reason, solve problems, comprehend complex ideas, and learn quickly (Gottfredson, 1997). It predicts numerous important life outcomes, including academic achievement (Lubinski, 2004; Roth et al., 2015), occupational success, socioeconomic status, income (Batty et al., 2009; Gottfredson, 2004; Lubinski, 2004), health, and longevity (Batty et al., 2009; Gottfredson & Deary, 2004).

The concept of general intelligence was first introduced by Charles Spearman as a common factor explaining the positive manifold of cognitive test outcomes—psychometric *g* (Spearman, 1904). Since Spearman, research on intelligence structure has moved to hierarchical models, but the majority of these models still includes a general intelligence factor. The currently perhaps most influential intelligence model, the Cattell–Horn–Carroll (CHC) model (McGrew, 1997, 2009; Schneider & McGrew, 2018), assumes a three-stratum structure with narrow abilities at the bottom that are indicators of broad abilities (such as fluid reasoning, comprehension–knowledge, perceptual speed), which are in turn influenced by a general factor. Although the existence of a general factor is open to debate in the CHC taxonomy (e.g., McGrew, 2009), virtually all intelligence tests whose development was based on the CHC model—and almost all intelligence tests in general—include a Full-Scale IQ (FSIQ) as an indicator of general intelligence that typically is a composite score of many diverse or of all subtests from a test battery. To avoid an intertwining of the theoretical construct of general intelligence and its measurement, we refer to the theoretical construct as *general intelligence*, to the latent measure of general intelligence as *general factor*, and to a (unit-weighted) subtest composite intended to measure general intelligence as *general intelligence composite (GIC)*.

As most intelligence tests include a GIC, a major question in test construction concerns the determinants of a reliable and valid measurement of general intelligence. A recent study by Farmer et al. (2020) investigated such determinants by comparing the reliability and accuracy of different intelligence composites from two test batteries. They systematically varied the heterogeneity, general factor loadings (both separately and in combination), and number of subtests and found that, as a single criterion, high general factor loadings were more important than heterogeneity for an accurate composite. The most accurate composites were those derived from numerous (12 to 13) diverse subtests with high general factor loadings, but the gains in reliability and accuracy began to flatten out from about four subtests on. Yet as the authors pointed out, small gains in reliability are of practical relevance, as they can have substantial effects on confidence intervals (CIs) and hence on comparability on an individual level (i.e., overlap of CIs). It is therefore important to investigate individual-level in addition to group-level comparability to learn more about the accuracy of different composites.

There are different kinds of score comparability or score linking, all of which technically require the application of a specific linking function (Dorans & Holland, 2000; Holland, 2007). However, the composite scores from intelligence tests are seldom linked directly using an explicit linking function. Rather, the different composites are standardized separately and it is presumed that different composites intended to measure the same construct, for example, general intelligence, will be *equal* or *exchangeable*. At least this is how these scores are applied in practice, where often one test is selected from a variety of different tests purporting to measure the same construct(s) and the resulting test score is interpreted as if it would have been the same (or at least very similar, considering measurement error) on any of the other tests. However, for scores to be regarded equal, at least five requirements have to be fulfilled that not necessarily hold for different GICs: (a) the same construct requirement, (b) the equal reliability requirement, (c) the symmetry requirement, (d) the equity requirement, and (e) the population invariance requirement (Dorans & Holland, 2000).

The same construct requirement holds that two tests need to measure the same theoretical construct, which requires this to be carefully defined based on a sound theory providing clear guidance for test development at the item level (Beaujean & Benson, 2019; Maul et al., 2019). We would assume that most concurrent intelligence tests will fail this requirement, at least for GICs. The equal reliability requirement is also often violated, especially when comparing scales of different length, but violations of this requirement are less important if reliabilities are high (Dorans & Holland, 2000). For intelligence tests, internal consistencies are usually very high and unreliability is often addressed using CIs. However, the question remains whether internal consistencies sufficiently capture the tests’ measurement error (see below). The symmetry requirement is usually met by definition (Dorans & Holland, 2000) and therefore not of interest for our study. The equity requirement concerns the exchangeability of test results and holds that an individual test outcome should be the same no matter which of the compared tests is used. It is this requirement that prior studies on individual-level comparability of GICs were most concerned with and that we mostly focus on in our study as well. Finally, the population invariance requirement holds that the compared scores should be equally comparable across all different (sub)populations the tests are intended for use. Violations of this requirement can be indicative of violations of the same construct and/or the equal reliability requirements (Dorans & Holland, 2000). It is tested by comparing the comparability of test results across specific subgroups of the whole population, which is what we did in the present study. To clarify whether it is justified to regard different GICs to be equal in the sense of Dorans and Holland (2000)—henceforth called *comparable*—it is thus important to investigate the degree to which the aforementioned requirements are fulfilled for these GICs.

The few studies we know of that dealt with the comparability of GICs in this sense performed individual-level comparisons between GICs derived from different test batteries (Bünger et al., 2021; Floyd et al., 2008; Hagmann-von Arx et al., 2018). These revealed substantial intraindividual absolute differences in GICs on an IQ scale—henceforth called IQ differences—and limited comparability of CIs and IQs in nominal categories. All three of the aforementioned studies concluded that any two intelligence tests do not necessarily render comparable FSIQs on the individual level, even if they show high correlations and no mean differences on the group level. Results from all three studies thus indicate violations of the equity requirement.

We add to these previous findings with the present study, in which we investigated the individual-level internal comparability of different GICs, that is, of different composites derived from the *same* test battery that are all intended to measure general intelligence. Proceeding this way, transient error (i.e., error due to variations in mood, information-processing efficiency, etc. over time; see Schmidt et al., 2003) as well as differences in examiner influences are kept to a minimum as all scores stem from a single test session, and between-battery variance (i.e., the standardization sample and differences in global test characteristic, such as general instructions, type of presentation) is held constant. Internal comparability analyses also have the advantage that they practically eliminate carryover effects, that is, the influence of practice effects on scores on a second test if this includes very similar tasks to the first test. For the purpose of internal comparability analyses, the comparison between the FSIQ and an Abbreviated Battery IQ (ABIQ) from the same test battery is well-suited. While the FSIQ is typically based on many or all subtests, an ABIQ is based on a subset of subtests and is thus typically less reliable than the FSIQ and intended as a screening. After practitioners have administered an ABIQ, their decision as to whether the rest of the test battery will also be administered is often based on the screening results (Thompson et al., 2004). It is therefore important to investigate the individual-level comparability of FSIQ and ABIQ.

In the present study, we examined this internal comparability of GICs—mainly FSIQ and ABIQ—in four steps. First, we determined the magnitude of intraindividual IQ differences between the GICs; second, we investigated the impact of these differences by comparing the GICs’ external validity; third, we examined possible predictors of these differences; and fourth, we sought ways to deal with incomparability.

Given imperfect reliability and results from Floyd et al. (2008), Hagmann-von Arx et al. (2018), and Bünger et al. (2021), we expected to find at least some IQ differences. To examine the possible impact of such differences on external validity, we determined the GICs’ differential relationships with school grades. As general intelligence measures are strong predictors of scholastic achievement and academic success (Deary et al., 2007; Gygi et al., 2017; Roth et al., 2015; Watkins et al., 2007), these criteria are typically used for external validation of intelligence tests. In our study, we focused not on the absolute magnitude of relationships between GICs and school grades but rather on possible differences in magnitude of relationships between the FSIQ and school grades and the ABIQ and school grades. While the former has been studied extensively (see above), to our knowledge, effect sizes of the FSIQ and the ABIQ have never been compared explicitly, which is what we did in the present study.

After having determined the magnitude and impact of IQ differences, we were interested in possible predictors of these. Results from previous studies suggest that most of the error variance in IQs is systematic (Irby & Floyd, 2016, 2017). To learn more about the sources of systematic variation in IQ differences, we explored several possible predictors of IQ differences. These include variables already considered in previous studies (Bünger et al., 2021; Hagmann-von Arx et al., 2018), such as IQ level and age, as well as other, not yet examined characteristics of the testee and their behavior in the test situation. If characteristics of the testee should explain some variation in IQ differences, this would indicate a failure of the population invariance requirement. Characteristics of the composite, such as the number, general factor loadings, and content of subtests involved, might also predict IQ differences, as these characteristics influence the accuracy of composites (see Farmer et al., 2020). Because these characteristics are invariant between individuals, inclusion in quantitative analyses was not possible here. Hence, we address them in a descriptive manner only.

As a last step, we explored possible ways to deal with incomparability. To this end, we examined alternative ways of describing intelligence test results other than exact IQ scores, aiming to achieve more reliable and stable estimates that may meet the equality requirements to a greater extent. Obvious candidates that were also examined in previous studies (Bünger et al., 2021; Floyd et al., 2008; Hagmann-von Arx et al., 2018) are CIs and nominal categories (e.g., “average” for an IQ between 85 and 115). In all three studies mentioned above, however, CIs were computed solely on the basis of an overall internal consistency, which reflects the most common use in practice. Results from these studies suggest that using such CIs still does not lead to satisfactory comparability. As an extension to these previous studies, we therefore varied the reliability coefficients used for the calculation of CIs. It is known that test–retest reliability tends to be lower at younger ages (Watkins & Smith, 2013) and toward the tails of the IQ distribution (due to regression to the mean; Campbell & Kenny, 1999). Considering floor and ceiling effects, the same might also be true for internal consistency. If this were the case, using CIs based on separate internal consistency coefficients for age and IQ groups—henceforth, called age- and IQ-specific internal consistencies—should lead to higher rates of comparability between IQs compared with using CIs based on the same overall internal consistency for all participants. This assumption is supported by results from Bünger et al. (2021), who found that IQ level was a significant predictor of IQ differences. A possible influence of age on comparability was not investigated by Floyd et al. (2008) and Hagmann-von Arx et al. (2018), and age was no systematic predictor for IQ differences in regression analyses reported in Bünger et al. (2021). However, as Bünger et al. (2021) concluded, further analyses with larger age groups are warranted to learn more about IQ comparability across age, which was possible in the present study. Hence, we investigated comparability across IQ level and age for all criteria, and we examined comparability for CIs based on age- and IQ-specific internal consistencies. Moreover, using internal consistency as a reliability estimate bears the danger of overestimating reliability, as it misses transient error (see above; Schmidt et al., 2003). This source of error is, however, assessed in test–retest reliability, which is why we also considered CIs based on test–retest reliability coefficients in our study.

## Present Study

The primary objective of this study was to investigate the individual-level internal comparability of different GICs. For this purpose, we compared GICs (mostly FSIQ vs. ABIQ) derived from the same test battery for participants from the standardization samples of three test batteries, spanning from early childhood to late adulthood: The *Intelligence and Development Scales–2* (IDS-2; Grob & Hagmann-von Arx, 2018a), and the German adaptations of the *Stanford–Binet Intelligence Scales–Fifth edition* (SB5; Grob et al., 2019b) and the *Reynolds Intellectual Assessment Scales* (RIAS; Hagmann-von Arx & Grob, 2014a). Since comparisons of GICs from different intelligence tests were not an aim of this study, we exclusively compared GICs *within* these test batteries. To learn more about the impact of, possible predictors for, and ways to deal with incomparability, secondary objectives of this study were to examine the differential external validity of GICs, to identify predictors of IQ differences, and to see if individual-level comparability could be enhanced by varying reliability coefficients for the calculation of 95% CIs.

We addressed the following hypotheses and research questions: First, we expected that (a) the GICs for each test battery would be highly intercorrelated, and (b) there would be no significant mean differences between GICs. Second, we examined the magnitude of intraindividual differences in IQ points (both overall and across IQ level and age). Third, we hypothesized that relationships of school grades with the ABIQ would be smaller compared with those with the FSIQ. Fourth, we examined whether certain characteristics of the testee (e.g., age) or the their behavior in the test situation (e.g., understanding of instructions) were associated with IQ differences. Finally, we examined how many participants would achieve comparable intelligence estimates (again both overall and across IQ level and age) determined with different criteria (i.e., different 95% CIs and nominal categories). We expected higher comparability for CIs based on age- and IQ-specific internal consistencies and test–retest reliabilities compared with CIs based on one overall internal consistency coefficient. Supplementary material (available online) to this study, including analysis scripts, is available at https://osf.io/hfqe5/.

## Method

### Participants

The IDS-2 standardization sample consists of 1,672 participants from Switzerland, Germany, and Austria. Complete data on all GICs were available for 1,622 participants (50.9% girls and women; age in years: *M* = 12.06, *SD* = 4.40, range: 5.02-20.97). About one third (31.4%) of participants’ mothers had a university degree, 16.5% of participants were bilingual (German and at least one other native language), 7.6% were nonnative speakers (German not their native language), and 3.4% reported having an attention-deficit/hyperactivity disorder (ADHD) or attention-deficit disorder (ADD) diagnosis (hereafter called AD[H]D). For a subsample of 414 individuals (50.7% girls and women; age in years: *M* = 12.07, *SD* = 2.59, range: 5.42–19.37), there were additional cross-sectional data on school grades.

The SB5 standardization sample consists of 1,829 participants from Switzerland, Germany, Austria, and Liechtenstein. Complete data on all GICs were available for all 1,829 participants (51.4% girls and women; age in years: *M* = 23.46, *SD* = 20.02, range: 4.00-83.96). Around one third (29.4%) of participants—or for children and adolescents, their mothers—had a university degree, 8.8% of participants were bilingual (German and at least one other native language), 7.8% were nonnative speakers (German not their native language), and 2.9% reported having an AD(H)D diagnosis. For a subsample of 249 individuals (47.4% girls; age in years: *M* = 11.31, *SD* = 2.38, range: 5.79–17.68), there were additional cross-sectional data on school grades.

The RIAS standardization sample consists of 2,145 participants from Switzerland and Germany. Complete data on all GICs were available for 2,109 participants (49.5% girls and women; age in years: *M* = 19.84, *SD* = 20.28, range: 3.00-99.96). About one fifth (20.7%) of participants—or for children and adolescents, their mothers—had a university degree, and 17.9% of participants were nonnative speakers (German not their native language). For a subsample of 64 individuals, there were additional data on school grades collected 2 to 4 years after the intelligence assessment (51.6% girls; age in years at T1: *M* = 9.02, *SD* = 1.02, range: 6.07-11.22, and at T2: *M* = 11.41, *SD* = 0.99, range: 9.00-14.00).

### Materials

#### Intelligence Test Batteries

The IDS-2 assess cognitive (intelligence, executive functions) as well as developmental (psychomotor skills, socioemotional skills, basic skills, and motivation and attitude) in 5- to 20-year-olds with a total of 30 subtests (Grob & Hagmann-von Arx, 2018a; see Table S1 in the online supplement material, for descriptions). The IDS-2 allow for the estimation of three different GICs. The Profile IQ (an Extended Battery IQ, henceforth called IDS-2_EBIQ(14)_) is based on all 14 subtests that also constitute a profile of the following seven broad abilities, each estimated by two subtests: Visual Processing, Processing Speed, Auditory Short-Term Memory, Visual-Spatial Short-Term Memory, Long-Term Memory, Abstract Reasoning, and Verbal Reasoning. The first seven subtests (one per broad ability) constitute a GIC without a factor profile–the FSIQ (IDS-2_FSIQ(7)_). Additionally, the two subtests with the highest general factor loadings in a confirmatory factor analysis of the first seven subtests—Completing Matrices and Naming Categories—constitute the ABIQ (IDS-2_ABIQ(2)_).^1^ Finally, the IDS-2 include a rating of the participation of the testee during testing with 12 questions answered by the test administrator at the end of the intelligence, executive functions, and developmental functions assessments. Here, we used the answers on the intelligence assessment only.

The SB5 are an intelligence test battery for 4- to 83-year-olds that include a total of 10 subtests (Grob et al., 2019b; see Table S1 in the online supplement material, for descriptions). The following five broad abilities can be estimated based on one verbal and one nonverbal subtest each: Fluid Reasoning, Knowledge, Quantitative Reasoning, Visual-Spatial Processing, and Working Memory. Additionally, the five verbal and five nonverbal subtests are used for a Verbal and a Nonverbal IQ. All 10 subtests are used for an FSIQ (SB5_FSIQ(10)_) and the two routing subtests—Nonverbal Fluid Reasoning and Verbal Knowledge—constitute the ABIQ (SB5_ABIQ(2)_). Finally, the SB5 include a rating of the participant’s understanding of instructions and cooperation in the test situation with one question each answered by the test administrator at the end of the test session.

The RIAS measure verbal and nonverbal intellectual abilities as well as memory with two subtests each (six in total) in 3- to 99-year-olds (Hagmann-von Arx & Grob, 2014a; see Table S1 for descriptions). The two corresponding subtests are used to form a Verbal Intelligence Index, a Nonverbal Intelligence Index, and a Memory Index. All four intelligence subtests are used for an FSIQ (RIAS_FSIQ(4)_). Additionally, one verbal and one nonverbal intelligence subtest—Guess What and Odd-Item Out—constitute the Reynolds Intellectual Screening Test (RIST; hereafter called the RIAS_ABIQ(2)_).

Having seven available GICs thus enabled five within-test battery comparisons: three for the IDS-2, one for the SB5, and one for the RIAS. The FSIQs from all three test batteries differ from each other in terms of number and content of subtests, whereas all ABIQs consist of one subtest each measuring fluid reasoning and comprehension knowledge (see Table 1). For the IDS-2_EBIQ(14)_ and IDS-2_FSIQ(7)_, as well as for the RIAS_FSIQ(4)_ and RIAS_ABIQ(2)_, only the number of subtests, and not the content, differs, as the corresponding GICs tap the same broad abilities in equal shares. In contrast, for the IDS-2_EBIQ(14)_/IDS-2_FSIQ(7)_ and IDS-2_ABIQ(2)_, as well as for the SB5_FSIQ(10)_ and SB5_ABIQ(2)_, content and number of subtests differ.

**Table 1.** Number, Position, and Content of Subtests, and Reliabilities and Widths of 95% CIs for each GIC.

GIC	# of Subt.	Pos. in Test Seq.	Content Overlap (%)	CHC Broad Abilities Tapped	Internal consistency			rtt^a^	Width of 95% CI			
					Overall^a^	Age-specific^a^	Age- and IQ-specific^b^		95% CI	95% CI_age_	95% CI_ageIQ_	95% CI_rtt_
IDS-2_EBIQ(14)_	14	1-14	100	Gf, Gc, Gsm, Gv, Glr, Gs	.98	.95-.97	.67-.98	.85	8	10-13	8-28	21
IDS-2_FSIQ(7)_	7	1-7	44	Gf, Gc, Gsm, Gv, Glr, Gs	.97	.92-.95	.54-.97	.89	10	12-16	10-30	19
IDS-2_ABIQ(2)_	2	6, 7	38	Gf, Gc	.95	.83-.90	.53-.92	.86	13	17-23	15-30	21
SB5_FSIQ(10)_	10	1-10	50	Gf, Gc, Gsm, Gv, Gq	.99	.93-.98	.45-.96	.94	6	8-16	10-30	14
SB5_ABIQ(2)_	2	1, 2		Gf, Gc	.97	.76-.93	.30-.93	.86	10	15-26	15-30	21
RIAS_FSIQ(4)_	4	1-4	100	Gf, Gc	.95	.93-.97	.55-.96	.88	13	10-16	11-30	19
RIAS_ABIQ(2)_	2	1, 2		Gf, Gc	.93	.90-.94	.51-.96	.87	15	13-18	12-30	20

*Note*. Content overlap was calculated by dividing the number of subtests tapping the same broad abilities for both GICs by the total number of subtests over both GICs and multiplying this decimal by 100. Each content overlap percentage concerns the respective GIC and the one in the row below (for the IDS-2_ABIQ(2)_: the IDS-2_EBIQ(14)_). CHC broad ability assignments are based on information in the test manuals and descriptions in McGrew (2009, Table 1). Mean test–retest intervals were 24 days (IDS-2), 22 days (SB5), and 19 days (RIAS). Age- and IQ-specific internal consistencies: IQ groups: <85, 85-115, >115; age groups: IDS-2: 5-6, 7-8, 9-12, 13-15, 16-20 years, SB5: <7, 7-8, 9-12, 13-15, 16-–20, 21-29, 30-59, ≥60 years, RIAS: 3-4, 5-6, 7-8, 9-12, 13-15, 16-20, 21-59, ≥60 years. GIC = general intelligence composite; CI = confidence interval; # of Subt. = number of subtests; Pos. in Test Seq. = position in test sequence; CHC = Cattell–Horn–Carroll; rtt = test–retest reliability; 95% CI = 95% CI with overall internal consistencies; 95% CI_age_ = 95% CI with age-specific internal consistencies; 95% CI_ageIQ_ = 95% CI with age- and IQ-specific internal consistencies; 95% CI_rtt_ = 95% CI with test–retest reliability; EBIQ = Extended Battery IQ; FSIQ = Full-Scale IQ; ABIQ = Abbreviated Battery IQ; IDS-2 = Intelligence and Development Scales–2; SB5 = Stanford–Binet Intelligence Scales–Fifth edition, German adaptation; RIAS = Reynolds Intellectual Assessment Scales, German adaptation; Gf = fluid reasoning; Gc = comprehension knowledge; Gsm = short-term memory; Gv = visual processing; Glr = long-term memory and retrieval; Gs = cognitive processing speed; Gq = quantitative knowledge.

a Derived from manuals. Based on Cronbach’s alphas (IDS-2 and RIAS) or split-half reliabilities (SB5).

b Computed according to the manuals with a formula provided by Lienert and Raatz (1998, p. 330) based on Cronbach’s alphas (IDS-2 and RIAS) or split-half reliabilities (SB5).

#### Participant and Parent Questionnaires

Adolescent and adult participants and/or—for children and adolescents—their parents reported on demographic variables, including age, sex, education (additionally for children and adolescents: education of the parents), native language, and psychological and physical abnormalities (including AD[H]D). In an additional questionnaire, some parents reported their child’s school grades in German (instructional language), mathematics, social studies, geography and history (combined), and science from the last two school semesters.

### Procedure

Participants were recruited through schools and psychosocial institutions for children and adolescents in Switzerland, Germany, and Austria. For the IDS-2, administration of the whole test battery took between 3 and 4 hours and, if necessary, could be split into two sessions not more than 1 week apart. Administration of the intelligence part alone took approximately 1.5 hours and was completed within one test session. For the SB5, administration took 1.5 to 2 hours and for the RIAS it took around 30 to 40 min. Written consent was obtained from children and adolescents (10 years and older) and/or from their parents (5- to 15-year-olds). The demographic questionnaire was administered at the beginning of the first session. The parental report of school grades was completed at home either within weeks after the session (IDS-2 and SB5) or as part of a follow-up study 2 to 4 years after the intelligence assessment (RIAS). Participants from Switzerland received a gift card of their own choice worth 30 (IDS-2) or 20 (SB5 and RIAS) Swiss francs and participants from Germany and Austria received 25 (IDS-2) or 12 (SB5 and RIAS) Euros in cash for participation.

### Statistical Analyses

All analyses were conducted in R (R Core Team, 2020). The complete analysis code is available at https://osf.io/hfqe5/. Within each test battery, we first inspected group-level comparability of GICs with Pearson correlations (both uncorrected and corrected for unreliability of both GICs) and paired samples *t* tests. For individual-level comparability, we then calculated intraindividual absolute differences in IQ points. To compare the GICs’ external validity, we performed linear regressions of school grades on GICs. All grades were transformed into Swiss school grades, ranging from 1 (*lowest*) to 6 (*highest*). In our study, we focused on grades in German and mathematics as well as on the grade point average (GPA). The GPA was computed as the average of all reported grades for each participant. As the GICs were expected to be highly correlated, we included them in separate models and compared the resulting *R*^2^s and 95% CIs for standardized regression coefficients (betas). Because the models were not nested, we could not determine the significance of the change in *R*^2^. Instead, following Cumming (2009), we regarded two betas as significantly different from one another if their 95% CIs overlapped to a degree of 50% or less.

We explored several possible predictors of IQ differences, specifically, age, sex, AD(H)D (yes vs. no), native language (monolingual German [reference] vs. bilingual and vs. other native language), IQ level (average [85 ≤ IQ ≤ 115, reference] vs. below average [IQ < 85] and vs. above average [IQ > 115]), education of the participant or—for children and adolescents—of their mother (university degree vs. no university degree), participation in the test situation (for IDS-2; age-standardized scores with *M* = 10 and *SD* = 3), cooperation in the test situation (for SB5; yes vs. no/partly), understanding of instructions (for SB5; yes vs. no/partly), and the interaction between IQ level and age. We used gamma generalized linear models with a log link function to model IQ differences. In contrast to a classic linear regression, with a normally distributed dependent variable (Gaussian family) and an identity link function (*g*[u] = u), the generalized linear models we used model a gamma-distributed dependent variable (Gamma family) with a log link function (*g*[u] = log[u]; see, e.g., McElreath, 2015, for more information on generalized linear models). Using such gamma generalized linear models, we could best account for the strongly right-skewed, nonnegative and continuous distribution of the dependent variables of absolute IQ differences (see Figure S1 in the online supplemental material). Following suggestions from Gelman (2008), we standardized all predictor variables by dividing by 2 *SDs*. This way, regression coefficients are directly comparable in size between continuous and binary predictors. We deemed an effect significant if both the overall model (determined with a likelihood ratio test) and the predictor were significant at an alpha level of .05. To illustrate the variation of IQ differences across IQ level and age, we compared the resulting difference scores across IQ level (including six IQ groups: < 70, 70-84, 85-99, 100-114, 115-129, ≥130; see Figure S3 in the online supplemental material) and across age (including different age groups depending on the test battery; see Figure S4 in the online supplemental material). The IQ groups were based on the GIC with the largest number of subtests for each test battery (i.e., the IDS-2_EBIQ(14)_, SB5_FSIQ(10)_, and RIAS_FSIQ(4)_). The same GICs were used for the predictor of IQ level in regression analyses.

To explore ways to deal with incomparability, we computed 95% CIs using the standard error of estimate together with the estimated true score (Lord & Novick, 1968; see also Dudek, 1979). For each test battery, we then calculated the percentage of participants for whom the 95% CIs for the IQs overlapped. We varied the reliability coefficients used for the calculation of 95% CIs to investigate their influence on individual-level comparability. The 95% CIs were based on overall internal consistencies (95% CI; for IDS-2 and RIAS: Cronbach’s alphas and for SB5: split-half reliabilities), age-specific internal consistencies (95% CI_age_; see Table S8 in the online supplemental material, for age groups), and test–retest reliabilities (95% CI_rtt_) obtained from the test manuals (Grob et al., 2019a; Grob & Hagmann-von Arx, 2018b; Hagmann-von Arx & Grob, 2014b; see Table 1 for reliabilities and CIs).

Additionally, we calculated 95% CIs based on age- and IQ-specific internal consistencies according to the manuals using a formula provided by Lienert and Raatz (1998, p. 330; 95% CI_ageIQ_; e.g., for 5- to 6-year-olds with IQ < 85; see Table 1 for IQ and age groups). Finally, we investigated the comparability of the IQs’ corresponding nominal categories (NomIQ; < 70 = lower extreme, 70-84 = below average, 85-115 = average, 116-130 = above average, >130 = upper extreme; see also Grob et al., 2013) as well as the comparability of the 95% CIs with overall internal consistencies in nominal categories (NomCI; e.g., average to above average for an interval of 112 to 120). For each of these six resulting criteria—95% CI, 95% CI_age_, 95% CI_ageIQ_, 95% CI_rtt_, NomIQ, and NomCI—two IQs were deemed comparable on an individual level if their intervals overlapped. Just as for IQ differences, we compared the percentages of participants with overlapping intervals across IQ level and age using the same groups.

## Results

### Group-Level Analyses

The seven GICs considered were normally distributed; their means were close to 100 (99.53 to 100.11) and standard deviations close to 15 (14.49 to 15.11, see Table 2). The IDS-2_FSIQ(7)_ had the narrowest range with 55 to 142, and the RIAS_ABIQ(2)_ had the widest range with 40 to 160. We compared the GICs within each test battery using *t* tests and Pearson correlations and found very small mean differences that were nonsignificant in all but one case (*d* = −0.002 for the IDS-2_FSIQ(7)_ vs. the IDS-2_ABIQ(2)_ to *d* = 0.031 for the RIAS_FSIQ(4)_ vs. the RIAS_ABIQ(2)_; the latter being significant, *t*(2108) = 3.73, *p* < .001). Intercorrelations both uncorrected and corrected for unreliability of both IQs were all significant and high to very high (*r* = .76 for the SB5_FSIQ(10)_ and the SB5_ABIQ(2)_ to *r* = .95 for the IDS-2_EBIQ(14)_ and the IDS-2_FSIQ(7)_, and *r_corr_* = .77 for the SB5_FSIQ(10)_ and the SB5_ABIQ(2)_ to *r_corr_* = .99 for the RIAS_FSIQ(4)_ and the RIAS_ABIQ(2)_, all with *p* < .001).

**Table 2.** Descriptive Statistics, Paired-Samples *t* Tests and Pearson Correlations, and Intraindividual Absolute Differences in IQs.

GIC	*M*	*SD*	Range	Skewness	Kurtosis	*t*	Cohen’s *d*	*r*	*r_corr_*	*M_diff_*	*Md_diff_*	Range_diff_
IDS-2_EBIQ(14)_	100.04	14.70	55-145	−0.49	0.65	−0.61	−0.01	.95***	.98***	3.68	3	0-20
IDS-2_FSIQ(7)_	100.11	14.79	55-142	−0.44	0.48	−0.12	−0.00	.82***	.86***	7.00	6	0-39
IDS-2_ABIQ(2)_	100.08	15.11	55-144	−0.30	0.10	−0.12	−0.00	.77***	.80***	7.94	7	0-37
SB5_FSIQ(10)_	99.96	14.80	55-145	−0.02	0.22	−0.18	−0.00	.76***	.77***	8.12	7	0-38
SB5_ABIQ(2)_	99.92	14.95	55-145	−0.06	0.00							
RIAS_FSIQ(4)_	99.53	14.77	45-158	−0.49	0.91	3.73***	0.03	.93***	.99***	4.37	4	0-20
RIAS_ABIQ(2)_	99.98	14.49	40-160	−0.79	1.63							

*Note*. IDS-2: *n* = 1,622; SB5: *n* = 1,829; RIAS: *n* = 2,109. The last six columns refer to the comparison between the respective GIC and the one in the row below it (for the IDS-2_ABIQ(2)_: with the IDS-2_EBIQ(14)_). Cohen’s *d* was calculated using the formula from Dunlap et al. (1996) for paired samples. GIC = general intelligence composite; EBIQ = Extended Battery IQ; FSIQ = Full-Scale IQ; ABIQ = Abbreviated Battery IQ; IDS-2 = Intelligence and Development Scales–2; SB5 = Stanford–Binet Intelligence Scales–Fifth Edition, German adaptation; RIAS = Reynolds Intellectual Assessment Scales, German adaptation; *r_corr_* = Pearson correlation corrected for unreliability of both GICs; *M_diff_*/*Md_diff_*/Range_diff_ *=* mean/median/range of intraindividual absolute IQ difference.

*** *p* < .001.

**Table 3.** Gamma Generalized Linear Models With Possible Predictors of Absolute Differences in IQs.

Predictor	IDS-2	SB5	RIAS
	EBIQ vs. ABIQ	FSIQ vs. ABIQ	FSIQ vs. ABIQ
Age	−0.00	−0.15**	−0.08*
Sex	0.00	0.03	0.03
AD(H)D	0.12	0.14	
*Native language*			
Bilingual	−0.14	0.00	−0.01
Other language	−0.04**	0.07	−0.01
Education	0.06	0.05	0.05
IQ level			
Below-average IQ	0.01***	0.07	0.27***
Above-average IQ	0.06	0.26	0.17**
Participation	0.00		
Cooperation		0.05	
Understanding		0.05	
Age * Below-average IQ	−0.59***	0.12	−0.07
Age * Above-average IQ	−0.10	0.31**	−0.16
Likelihood	24.04*	27.45**	29.52***

*Note*. IDS-2: *n* = 1,566, SB5: *n* = 1,775, RIAS: *n* = 1,979. Displayed are regression coefficients standardized by dividing by two standard deviations (Gelman, 2008). Sex: 0 = male, 1 = female; AD(H)D: 0 = no, 1 = yes; Bilingual: 0 = German, 1 = bilingual; Other language: 0 = German, 1 = other native language; Education (of participants or their mothers): 0 = no university degree; 1 = university degree; Below average IQ: 0 = 85 ≤ IQ ≤ 115, 1 = IQ < 85; Above average IQ: 0 = 85 ≤ IQ ≤ 115, 1 = IQ >115; Cooperation (in the test situation) and understanding (of instructions): 0 = yes, 1 = partly/no. AD(H)D = attention deficit/hyperactivity disorder or attention deficit disorder; Participation = participation in the test situation; IDS-2 = Intelligence and Development Scales–2; SB5 = Stanford–Binet Intelligence Scales–Fifth edition, German adaptation; RIAS = Reynolds Intellectual Assessment Scales, German adaptation; EBIQ = Extended Battery IQ; FSIQ = Full-Scale IQ; ABIQ = Abbreviated Battery IQ.

* *p* <.05. **p <.01. ***p <.001.

### Intraindividual Differences

The mean (and median) intraindividual absolute differences ranged between 3.68 (*Mdn* = 3) IQ points for the IDS-2_EBIQ(14)_ versus the IDS-2_FSIQ(7)_ and 8.12 (*Mdn* = 7) IQ points for the SB5_FSIQ(10)_ versus the SB5_ABIQ(2)_, with ranges between 0 and 20 (IDS-2_EBIQ(14)_ vs. IDS-2_FSIQ(7)_ and RIAS_FSIQ(4)_ vs. RIAS_ABIQ(2)_) and 0 and 39 IQ points (IDS-2_FSIQ(7)_ vs. IDS-2_ABIQ(2)_; see Table 2). The relative differences were normally distributed around 0 (see Figure S2). Absolute differences across IQ groups and age are displayed in Figures S3 and S4, respectively (see also Table S2). For most comparisons, differences tended to increase with higher IQs and for the IDS-2_EBIQ(14)_ versus the IDS-2_FSIQ(7)_ and the RIAS_FSIQ(4)_ versus the RIAS_ABIQ(2)_, they tended to decrease with lower IQs. Regarding age, differences were lowest for middle childhood for the SB5_FSIQ(10)_ versus the SB5_ABIQ(2)_, but highest for the same age period for the RIAS_FSIQ(4)_ versus the RIAS_ABIQ(2)_. Otherwise, differences showed little variation across age.

### Differential Relationships With School Grades

To compare the GICs’ external validity, we investigated their differential relationships with school grades in German and mathematics, and with the GPA. Comparisons of 95% CIs for the betas revealed that the relationship with the FSIQ was significantly higher than that with the ABIQ only for the SB5 and mathematics (see Figure S5 and Table S3).

**Figure 1.**
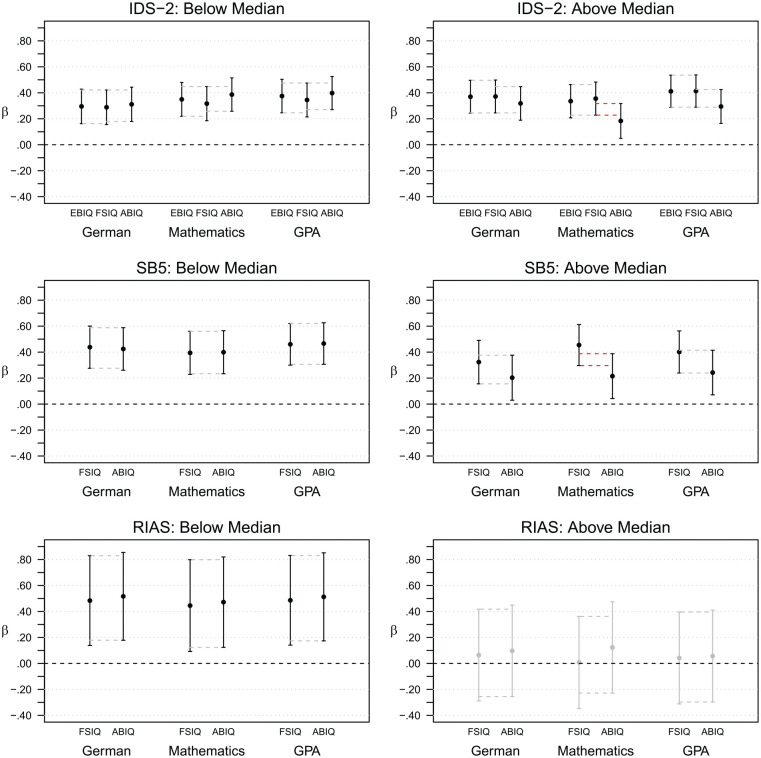
Comparison of 95% confidence intervals for standardized beta coefficients for GICs predicting school grades in German and mathematics and grade point average (GPA), split into subsamples with participants with intraindividual absolute differences in IQs below (IDS-2: *n* = 203, SB5: *n* = 122, RIAS: *n* = 29) and above (IDS-2: *n* = 211, SB5: *n* = 127, RIAS: *n* = 35) the median. *Note*. A difference in betas was deemed significant if confidence intervals overlapped to a maximum of 50% (indicated in red). Significant betas are in black, nonsignificant betas in gray. Data for the IDS-2 and SB5 are cross-sectional; data for the RIAS are longitudinal. IDS-2 = Intelligence and Development Scales–2; SB5 = Stanford–Binet Intelligence Scales–Fifth edition, German adaptation; RIAS = Reynolds Intellectual Assessment Scales, German adaptation; EBIQ = Extended Battery IQ; FSIQ = Full-Scale IQ; ABIQ = Abbreviated Battery IQ.

In a post hoc analysis, we repeated the external validity analyses for subsamples with small (below median) and large (above median) IQ differences to see how incomparability might affect external validity (see Figure 1 and Table S4). For individuals with small IQ differences, we found small to medium relationships that were all highly significant (β = .29 for the IDS-2_FSIQ(7)_ and German to β = .52 for the RIAS_ABIQ(2)_ and German, all with *p* < .001), and there were no significant differences in betas between the GICs. For individuals with large IQ differences, however, betas were still significant for the IDS-2 and SB5 (β = .18, *p* = .008 for the IDS-2_ABIQ(2)_ and mathematics to β = .46, *p* < .001 for SB5_FSIQ(10)_ and mathematics), but lower for the SB5 and no longer significant for the RIAS (β = .01, *p* = .965 for the RIAS_FSIQ(4)_ and mathematics to β = .12, *p* = .483 for the RIAS_ABIQ(2)_ and mathematics). For the IDS-2 and SB5, relationships with the ABIQ were also consistently smaller compared with those with the FSIQ and the EBIQ, although for both, this difference in betas was only significant for mathematics (see Figure 1).

### Possible Predictors of IQ Differences

Next, we investigated possible predictors of IQ differences. Only the models for the comparisons of the IDS-2_EBIQ(14)_ versus the IDS-2_ABIQ(2)_, the SB5_FSIQ(10)_ versus the SB5_ABIQ(2)_, and the RIAS_FSIQ(4)_ versus the RIAS_ABIQ(2)_ were significant. Therein, IQ level and age and/or their interaction were the only consistent predictors (see Table 3; see Table S5 for results for all comparisons). Larger differences occurred for younger individuals for the SB5 and the RIAS, for individuals with a below-average IQ for the IDS-2 and the RIAS, and for individuals with an above-average IQ for the RIAS. Finally, there was a significant interaction effect for age and below-average IQ for the IDS-2 and for age and above-average IQ for the SB5 (see Figure S6). For the former, age was negatively associated with differences for individuals with below-average IQ, but not for individuals with average or above-average IQ. For the latter, although there was no main effect of IQ level, age was positively associated with differences for individuals with an above-average IQ, but negatively associated with differences for individuals with an average or below-average IQs (see the online supplementary material for a detailed description of results).

### Comparability Using Different Criteria

Table 1 shows the reliabilities and widths of the corresponding 95% CIs for all seven GICs. The width of the 95% CIs based on overall internal consistencies ranged between 6 (SB5_FSIQ(10)_) and 15 (RIAS_ABIQ(2)_) IQ points. Those based on age-specific internal consistencies and test–retest reliabilities were considerably larger, and those based on age- and IQ-specific internal consistencies reached up to 30 IQ points for some combinations of IQ > 115 and different age groups. The lowest age- and IQ-specific internal consistencies, resulting in the largest CIs, were found exclusively in groups with IQ > 115 and did not coincide with the lowest sample sizes for any of the IQs.

**Figure 2.**
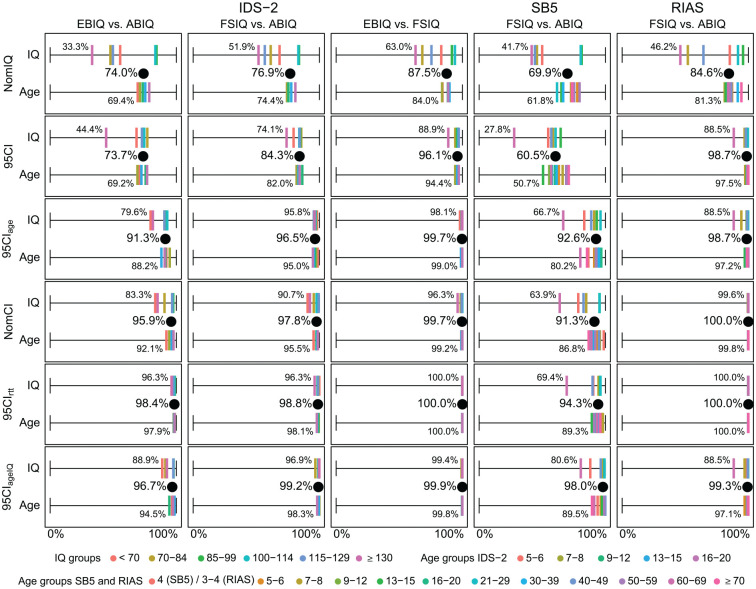
Percentage of participants with comparable IQs (i.e., overlapping intervals) determined by the following six criteria: NomIQ, 95% CI, 95% CI_age_, NomCI, 95% CI_rtt_, and 95% CI_ageIQ_. *Note*. NomIQ = IQ in nominal categories (e.g., “average” for IQ 85-115), 95% CI = 95% CI with overall internal consistencies, 95% CI_age_ = 95% CI with age-specific internal consistencies, NomCI = 95% CIs with overall internal consistencies in nominal categories, 95% CI_rtt_ = 95% CI with test–retest reliabilities, and 95% CI_ageIQ_ = 95% CI with age- and IQ-specific internal consistencies. The percentage of participants with comparable IQs both overall (black dots) and across IQ and age groups (color palette) are displayed. Numbers are displayed for the IQ and age group with the lowest percentage of participants with comparable IQs for each comparison. Ages given in years. IDS-2 = Intelligence and Development Scales–2; SB5 = Stanford–Binet Intelligence Scales–Fifth edition, German adaptation; RIAS = Reynolds Intellectual Assessment Scales, German adaptation; EBIQ = Extended Battery IQ; FSIQ = Full-Scale IQ; ABIQ = Abbreviated Battery IQ; CI = confidence interval.

The percentage of participants with comparable IQs (i.e., overlapping intervals) varied considerably across the different criteria and across IQ and age groups (see Figure 2 and Tables S6 to S11). With the 95% CI criterion, overall comparability was between 60.5% and 98.7%. Across IQ groups it ranged between 27.8% and 99.6% and across age groups between 50.7% and 100%. The overall comparability was lowest for the NomIQ (69.9% to 87.5%) and the 95% CI (60.5% to 98.7%) criteria and highest for the 95% CI_rtt_ (94.3% to 100.0%) and the 95% CI_ageIQ_ (96.7% to 99.9%) criteria. The same pattern was evident across IQ and age groups, with the lowest comparability for the NomIQ and the 95% CI and highest comparability for the 95% CI_rtt_ and the 95% CI_ageIQ_. In general, comparability was lowest for the comparison of the SB5_FSIQ(10)_ versus the SB5_ABIQ(2)_ and highest for the comparison of the RIAS_FSIQ(4)_ versus the RIAS_ABIQ(2)_.

## Discussion

The primary objective of this study was to investigate the individual-level internal comparability of different GICs. As expected, all GICs were highly intercorrelated and—with one exception—there were no significant mean differences. Despite this high correspondence on the group level, individual-level comparability was not always satisfactory. Intraindividual absolute differences reached up to 39 IQ points and tended to be larger for above-average IQ and younger ages. With respect to external validity, the EBIQ and FSIQ explained more variance in school grades compared with the ABIQ only for individuals with large IQ differences and only for the IDS-2 and SB5, with significant differences only for mathematics. Regarding possible predictors, IQ level and age, and/or their interaction, were the only consistent predictors of IQ differences. Finally, regarding ways to deal with incomparability, comparability varied considerably across criteria and again across both IQ level and age both within and between comparisons. While comparability for the NomIQ and 95% CI was often unsatisfactory, it was very high for the 95% CI_rtt_ and 95% CI_ageIQ_.

### Group-Level Comparability and Intraindividual Differences

On the group level, all GICs within each test battery were highly comparable, with the exception of the RIAS_FSIQ(4)_ and RIAS_ABIQ(2)_, where we found a significant mean difference despite a very high correlation. However, the effect size was very small, suggesting the effect is negligible. Despite high comparability on the group level, intraindividual absolute differences between GICs varied considerably, from 0 to more than 2.5 *SD*s (*M* between 0.25 and 0.53 *SD*s), depending on the comparison. There were no systematic differences in one direction; the relative differences were normally distributed around 0 for all comparisons. The mean IQ differences were slightly lower than those found in previous studies investigating individual-level comparability of FSIQs between test batteries (Bünger et al., 2021; Floyd et al., 2008; Hagmann-von Arx et al., 2018). Still, the size of the differences seems remarkable, given that the subtests used for the GICs overlap, that transient error is kept to a minimum, that the GICs were standardized on the same individuals, and that between-battery variance in general is ruled out completely. Revisiting the requirements introduced above that need to be fulfilled as a prerequisite for equal scores (Dorans & Holland, 2000), these findings indicate that the equity requirement is violated for the compared GICs, and thus the scores are not exchangeable.

### Differential External Validity

Analyses on differential external validity revealed that, as could be expected due to its lower reliability, the ABIQ tended to show weaker relationships with school grades compared with the FSIQ and EBIQ for most comparisons. However, this discrepancy in relationships with school grades was only significant for participants with large IQ differences and only for the IDS-2 and SB5 and mathematics. The two comparisons with the largest discrepancies also featured the largest IQ differences.

Apparently, the ABIQs miss aspects of intelligence that are contained in the EBIQ and FSIQs that are especially important for mathematical achievement. For the IDS-2, this probably concerns additional working memory aspects and visual–spatial skills known to be especially important for mathematical achievement (e.g., Bull & Lee, 2014; Kahl et al., 2021; McCrink & Opfer, 2014), that are included in the FSIQ and EBIQ, but not the ABIQ. For the SB5, the incremental validity of the FSIQ is probably mostly due to the quantitative knowledge subtests, and the working memory and visual–spatial processing subtests as well. Moreover, relationships were smaller for individuals with large compared with small IQ differences for the SB5 and RIAS, to the point that, for the RIAS (longitudinal analysis), they were no longer significant for individuals with large IQ differences.

From these findings, we conclude that a GIC based on more subtests is not necessarily a better predictor for school grades compared with one based on fewer subtests, especially for individuals with low IQ differences. We also conclude that larger IQ differences do have consequences for external validity, as the GICs for which larger intraindividual differences occurred were also the ones with larger disparities in relationships with school grades, and as relationships tended to be lower in general for individuals with high IQ differences. Finally, differences in content seem to be more important than differences in the number of subtests per se for differential external validity.

### Possible Predictors of IQ Differences

Given results from previous studies showing that most error variance in IQs was systematic (Floyd et al., 2008; Irby & Floyd, 2016, 2017), it is likely that the IQ differences we found are not entirely due to random error. Our results suggest that characteristics of the testee are likely one systematic influence, as IQ differences varied across IQ level and age, and those two and/or their interaction were the only systematic predictors in regression analyses. These results are in line with Hagmann-von Arx et al. (2018) and Bünger et al. (2021), where, for some comparisons, IQ differences were larger for younger participants and at the tails of the IQ distribution as well.

With respect to age, younger participants had higher IQ differences for the SB5 and the RIAS (age range: early childhood to late adulthood) but not for the IDS-2 (age range: early childhood to late adolescence). In Bünger et al. (2021), age was also not systematically linked to IQ differences, indicating that the effect of age might also depend on individual test characteristics. With respect to IQ level, the finding of larger differences toward the tails of the IQ distribution is to be expected due to regression to the mean (Campbell & Kenny, 1999). In Hagmann-von Arx et al. (2018), IQ level was a significant predictor of IQ differences also only for some comparisons, while in Bünger et al. (2021), it was for all included comparisons. In both studies, all effects went in the direction of larger differences toward the tails of the IQ distribution as well.

Besides regression to the mean, floor and ceiling violations could also explain larger differences at the tails of the IQ distribution and at younger ages (e.g., Irby & Floyd, 2017). The raw scores are usually not scaled homogenously across the full spectrum of scores, such that small differences in the number of correct responses will have a disproportionate effect on scaled scores at the extremes of the ability spectrum (i.e., very high or very low ability, or very young age).^2^ This disproportionate influence at the extremes is more pronounced the fewer subtests/items are included in a composite, further questioning the use of really short measures (cf. Irby & Floyd, 2016, 2017).

A third and related explanation for larger differences toward the tails of the IQ distribution is the composite score extremity effect (Schneider, 2016), that is, the fact that a composite score tends to be more extreme than the average of the subtest scores it is composed of. This effect is larger the more subtests are included in a composite. Hence, a GIC composed of more subtests should render higher scores for above-average IQ, and lower scores for below-average IQ, compared with a GIC composed of less subtests. Table S12 illustrates this effect for our comparisons. However, this influence was less pronounced, as absolute IQ differences were not necessarily larger for comparisons of GICs with larger differences in the number of subtests (see below).

Fourth and last, larger IQ differences at the upper extreme of the IQ distribution are probably also in part due to Spearman’s law of diminishing returns (SLODR, Spearman, 1927). In line with SLODR, it has been shown that the general factor loadings of CHC broad ability factors decreased, and their specific variance increased with increasing IQ level (e.g., Reynolds, 2013; Tucker-Drob, 2009). Consequently, the validity of a GIC from the five broad ability composites also decreased with increasing IQ level. It can therefore be expected that GICs that sample different broad abilities (or the same, but to varying extents) will differ more for individuals with higher IQ. Thus, the effect of SLODR might cumulate with the aforementioned factors decreasing comparability at high IQ levels, and at the same time might diminish the effect of said other factors at low IQ levels. Our results of slightly larger differences at the upper tail of the IQ distribution compared with the lower tail support this notion.

In our study (and not investigated in previous studies) there were also significant interaction effects between IQ level and age. From the above considerations follows that the disproportionate influence of few items should be especially pronounced for older individuals with high IQ and for younger individuals with low IQ. Regression results support this in that the significant interaction effects went in the expected direction. All in all, our findings indicate that these two variables—IQ level and age—should be considered in conjunction with each other when calculating reliability coefficients.

Finally, the included predictors explained a significant amount of variance for only three of the five comparisons. It is likely that other variables that could not be sufficiently considered in the present study contribute to systematic variance in IQ differences, for example (achievement) motivation, attention span, or alertness.

Thus, there are at least two characteristics of the testee (i.e., IQ level and age) that explain some of the variance in IQ differences. These findings indicate that the population invariance requirement is violated, possibly due at least in part to violations of the same construct and equal reliability requirements (Dorans & Holland, 2000).

A second source of systematic variability, characteristics of the composites, likely played a role as well. Three such characteristics are number, general factor loadings, and content of subtests included in the composites. Farmer et al. (2020) showed that the most accurate composites are those derived from numerous (12 to 13) diverse subtests with high general factor loadings, where high general factor loadings are more important compared with heterogeneity. Their results also suggest that including fewer than four subtests results in substantial losses of accuracy. In line with common practice, the ABIQs included in our study are all composed of only two subtests. Furthermore, although all three ABIQs fulfill the heterogeneity criterion with the two subtests representing different broad abilities, only the subtests for the IDS-2_ABIQ(2)_ were chosen based on the highest general factor loadings. The SB5_ABIQ(2)_ is composed of the subtests with the lowest (Nonverbal Fluid Reasoning) and third lowest (Verbal Knowledge) general factor loading (Grob et al., 2019a), which might at least partly explain the larger differences we found for the SB5 compared with the IDS-2 and the RIAS.

Subtest content may also play a role. In this regard, it is especially interesting to compare the comparisons of IDS-2_EBIQ(14)_ versus IDS-2_FSIQ(7)_ and RIAS_FSIQ(4)_ versus RIAS_ABIQ(2)_. Both comparisons have the same degree of overlap in content (100%, see Table 1) and the same ratio of subtests (2:1) but different absolute numbers of subtests (4 and 2 vs. 14 and 7) and different numbers of broad abilities tapped (2 vs. 7). Differences for IDS-2_EBIQ(14)_ versus IDS-2_FSIQ(7)_ are slightly lower than for RIAS_FSIQ(4)_ versus RIAS_ABIQ(2)_, but both are considerably lower compared with the other comparisons.

To conclude, the same construct requirement is likely also violated, and larger overlap in content and high general factor loadings—thus, the fulfilment of the same construct requirement—seems to be more important than the sheer number of subtests for individual-level comparability. However, as our set of comparisons is very limited, these findings clearly need replication, ideally with comparisons of composites systematically varied in content, general factor loadings, and number of subtests.

### Ways to Deal With Incomparability

We explored several alternatives to exact IQ scores—namely, nominal categories and 95% CIs based on different reliability coefficients—with the aim of achieving a more dependable intelligence estimate. Results on percentages of participants with overlapping 95% CIs or nominal IQs reflect results on IQ differences in that they varied both between the different comparisons and across IQ level and age. Although all investigated criteria consider unreliability in some way, comparability still tended to be lower at younger ages and toward the tails of the IQ distribution.

Furthermore, comparability varied considerably between the different criteria. Although the overall percentages of participants with overlap of the 95% CI and the NomIQ tended to be higher compared with those found in previous studies on between-battery comparisons (Bünger et al., 2021; Hagmann-von Arx et al., 2018), they were still unsatisfactory. Especially when calculated separately for IQ and age groups, the percentage of participants with comparable IQs was sometimes very low, down to 28%. Rates of comparability were higher for the 95% CI_age_ and the NomCI criteria but the highest rates were achieved with the 95% CI_rtt_ or the 95% CI_ageIQ_ criteria. This is to be expected, given that the intervals were also often widest for these criteria. Which of the two—95% CI_rtt_ or 95% CI_ageIQ_—provides a better trade-off between comparability and precision (interval width) is difficult to pin down as this varies across GICs and across GIC comparisons. It is important to note here that we had to rely on fairly rough groups for IQ (<85, 85-115, and >115) and for age in adulthood (e.g., age 30-59 years for the SB5 and age 21-59 years for the RIAS). Additionally, group sizes varied considerably and were sometimes very low (IDS-2: *n* = 31 to *n* = 352; SB5: *n* = 15 to *n* = 222; RIAS: *n* = 23 to *n* = 175). The comparability versus precision trade-off could probably be improved for the 95% CI_ageIQ_ if larger, more fine-graded groups were considered, which would necessitate sampling more participants of diverse ages at the tails of the IQ distribution. Finally, both internal consistency and test–retest reliability miss certain kinds of measurement error. While internal consistency does not consider transient error, test–retest reliability does not consider specific factor error (i.e., errors due to individual interpretation of items; Schmidt et al., 2003). Therefore, other approaches may be even more beneficial. The coefficient of equivalence and stability (Cronbach, 1947), for example, considers both specific factor error and transient error. As this coefficient requires the administration of two parallel test forms on two different measurement occasions, we were not able to consider it in our study.

Finally, given the numerous equality requirements that are violated, more accurate CIs can be only part of the solution to incomparability, mainly as a means for practitioners to deal with incomparability of results from existing intelligence tests. Given the substantial differences we found, the consequences they have for validity, and the large intervals needed to achieve satisfactory individual-level comparability, the long-term goal must be to create more reliable and valid intelligence measures. To achieve a higher individual-level comparability, it might be necessary to question our current understanding of general intelligence and to refrain from multidimensional measures (i.e., subtests intended to measure both general intelligence and a broad ability; see also Beaujean & Benson, 2019). Instead, test developers could try to create unidimensional measures of specific broad abilities with a firmer theoretical and neurological basis (e.g., Beaujean & Benson, 2019; Kovacs & Conway, 2019). In this vein, using fluid reasoning measures instead of GICs composed of multiple broad abilities might be beneficial for diagnostic utility, especially at the upper end of the IQ distribution, as Reynolds (2013) found fluid reasoning to be the only composite not influenced by SLODR and being the best indicator for general intelligence across IQ levels and all investigated age levels (except 5-6 year-olds, where Comprehension–Knowledge was slightly better). For IQ > 115, it was even better than a GIC composed of all five broad abilities. More narrowly defined constructs and carefully developed, theory-driven instruments to measure these constructs as reliably and validly as possible are a prerequisite for the same construct requirement—and with this also the equity and population invariance requirements (Dorans & Holland, 2000)—to be fulfilled and for the interpretation of test results to have meaning beyond the particular test that was used.

### Implications

Our findings have implications for the construction, validation, and application of intelligence tests. First, they raise awareness that choosing the subtests with the highest general factor loadings for a short form does not necessarily result in comparable results to those for the full test battery. However, it is certainly better than choosing subtests with lower general factor loadings (see also Farmer et al., 2020).

Second, our results indicate that in terms of both individual-level comparability and external validity there are no large gains between the 7- and 14-subtest composites (the FSIQ and the EBIQ, respectively) for the IDS-2. In line with results from Farmer et al. (2020), this suggests a diminishing marginal utility of additional subtests—especially if they do not introduce other broad abilities—from a certain number of subtests on.

Third, our results speak against using one internal consistency coefficient derived from the whole sample for the calculation of CIs. Instead, we recommend the use of test–retest reliabilities, age- and IQ-specific internal consistencies or, probably even better, the coefficient of equivalence and stability (Schmidt et al., 2003). The additional resources spent on the construction and application of a parallel test form would be compensated for by more accurate reliability estimates and by a test battery that could be administered twice to the same testee without introducing learning effects. Ideally, the test–retest sample should also be large enough to permit at least a rough division into IQ and age groups to enable the use of age- and IQ-specific test–retest reliabilities or coefficients of equivalence and stability for the calculation of CIs.

Fourth, we encourage test developers to reconsider the current understanding of general intelligence, and to try to develop purer (i.e., unidimensional) measures guided by formal theories (e.g., Beaujean & Benson, 2019), as clearly defined constructs are a prerequisite for individual-level comparability of test scores.

Fifth, exact IQ scores should not be used for the interpretation or communication of test results. Indeed, in line with Bünger et al. (2021), our results show that even the 95% CI might not necessarily be valid enough for clinical interpretation, but it is certainly more appropriate than an exact IQ score. As done before (Bünger et al., 2021), we again call for a paradigm shift away from exact IQ scores toward intervals that consider the unreliability of intelligence composites in clinical interpretation. Instead of requiring an IQ score to fall above or below a certain threshold, the upper and lower levels of the 95% CI should be considered.

Sixth, our results demonstrate that the differences between the FSIQ and the ABIQ are largest especially in those ranges where most clinical questions arise—namely, at the tails of the IQ distribution. This is true even if 95% CIs are based on the expected true score, thus accounting for regression to the mean. To avoid the risk of missing diagnostically meaningful information, we suggest using a short test of at least four subtests (see Farmer et al., 2020) instead of an ABIQ with less subtests for screening purposes. A context, gaining importance in many Western countries, in which very short measures should be especially avoided, is for testees with low familiarity with (standardized) testing or test content as well as with difficulties in understanding task instructions. Following insights from dynamic testing (Beckmann, 2014; Beckmann & Dobat, 2000; Cho & Compton, 2015; Guthke & Wiedl, 1996), test performance for these testees increases in predictive validity with increasing time spent with the tasks. For example, it was shown that in a test–retest design, performance in the posttest was a better predictor for scholastic achievement compared with performance in the pretest, especially for disadvantaged children (Guthke & Wiedl, 1996). The use of a screening instrument thus bears the risk of underestimating an individual’s intellectual potential especially in these contexts.

Finally, IQ differences are linked to the prediction of school grades. For individuals with higher IQ differences, relationships with school grades tended to be lower in general, and especially for the ABIQ. In the long run, GICs might not even be predictive at all for school grades for these individuals. It is therefore important to identify these individuals, for example, through multiple testing, and to be aware of the possibility of reduced reliability and (external) validity of GICs in these cases.

Future research should determine to what extent the present results are applicable to broad ability composites as well. If two subtests are likely not enough for a GIC, this should be even less appropriate for a broad ability composite, given the small unique variance over and above the general factor such broad ability composites already capture (e.g., Cucina & Howardson, 2017). At the same time, content overlap should be larger for broad ability composites, raising the possibility of higher comparability of these scores compared with more heterogeneous GICs, at least after unreliability is taken into account. Interestingly, this is exactly what Bünger et al. (2021) found for verbal index scores from different intelligence test batteries. Comparability of CIs for broad ability composites reported in Floyd et al. (2005) also tended to be larger compared with the comparability of GICs reported in Bünger et al. (2021), Floyd et al. (2008), and Hagmann-von Arx et al. (2018), despite often larger absolute differences in IQ points for broad ability composites.

We also advocate the use of individual-level comparisons in addition to group-level analyses for validation of a test procedure intended for individual diagnostics. More research is needed to further investigate characteristics of both the testee and the test itself that are associated with individual-level incomparability of intelligence composites. Finally, in addition to internal and structural validation, a greater emphasis should be placed on external validation, but also on diagnostic and treatment utility, of test scores to determine their usefulness as a diagnostic instrument in practice.

### Strengths and Limitations

We investigated group- and individual-level internal comparability of GICs for a set of three test batteries based on large, representative samples covering a large age span from early childhood to late adulthood. In comparing GICs within test batteries, we were able to eliminate all kinds of variance between test batteries or test situations (including carryover effects, differences in standardization samples and global test characteristics, and transient errors), leaving characteristics of the testee and the test itself as the primary systematic sources of variance.

A limitation of this study is that we could form only broad IQ groups for age- and IQ-specific 95% CIs (i.e., below average, average, above average) due to small sample sizes within age groups. Greater oversampling of participants of different ages at the tails of the IQ distribution is needed to achieve more fine-graded groups and with this to ensure reliability and validity at the extremes.

Furthermore, we used school grades as a single criterion of external validity. Although school grades are strongly related to general intelligence (Roth et al., 2015), future research should consider differential relationships of GICs based on different numbers of subtests with additional criteria for scholastic achievement, such as scholastic aptitude tests or teacher ratings of school performance, as well as with criteria that are also valid for adults, for example, educational attainment or occupational success.

Finally, we could include only a limited number of test batteries and composites in our study. Systematic comparisons of the kind performed in Farmer et al. (2020)—comparisons of composites systematically varied in characteristics such as number, general factor loadings, and content of subtests—but on an individual level and within multiple test batteries are needed to further clarify the number and nature of subtests necessary to achieve more reliable and valid measures of general intelligence.

## Conclusion

Our findings raise awareness of the limitations of ABIQs as a means to get a first impression of an individual’s intellectual potential. Despite high comparability on the group level, individual-level comparability of GICs derived from the same test battery was often unsatisfactory. We therefore advocate to acknowledge a lower reliability of GICs to achieve more accurate intelligence assessments. One step in that direction would be to refrain from using internal consistencies and to instead use test–retest reliabilities or, probably even better, the coefficient of equivalence and stability (Cronbach, 1947) as a basis for CIs. The systematic effects of IQ level and age on IQ differences we found suggest that reliabilities should also be computed separately for age and IQ groups. Most important, our results demonstrate that the interpretation of exact IQ scores should be avoided. However, despite limited comparability with the FSIQ, we found that ABIQs did not necessarily display less external validity. But GICs in general, and especially ABIQs, tended to be worse predictors of school grades, especially in mathematics, for individuals with large intraindividual IQ differences.

To conclude, our results point to substantial intraindividual IQ differences that have consequences for external validity and are at least in part explained by IQ level and age. Our results demonstrate that a focus on CIs based on reasonable reliability coefficients is one way to deal with incomparability. Yet, further research is needed to learn more about the number and kind of subtests necessary to achieve an accurate measurement of general intelligence on the individual level.

## Notes

1 This distinction of an extended battery (in the IDS-2: 14 subtests), a standard battery (in the IDS-2: seven subtests), and an abbreviated battery (in the IDS-2: two subtests) is also made in other test batteries, for example, the Woodcock–Johnson IV (Schrank et al., 2014) and the Universal Nonverbal Intelligence Test 2 (Bracken & McCallum, 2016). The abbreviated battery is typically intended for screening purposes, the standard battery as an accurate and yet time efficient measure for diagnostic purposes, and the extended battery as a comprehensive measure that typically enables more or better defined subscale scores (e.g., Schrank et al., 2014) and/or a more reliable and valid measure of general intelligence to be used for high-stakes decisions (e.g., Bracken & McCallum, 2016).

2 For tests that only cover an age span in childhood, typically the same is true for individuals at the upper tail of the age distribution of the standardization sample. This was not the case for any of the test batteries in our study.
